# Telomere length and mortality in the Ludwigshafen Risk and Cardiovascular Health study

**DOI:** 10.1371/journal.pone.0198373

**Published:** 2018-06-19

**Authors:** Irene Pusceddu, Marcus Kleber, Graciela Delgado, Wolfgang Herrmann, Winfried März, Markus Herrmann

**Affiliations:** 1 Laboratory of Clinical Pathology, Hospital of Bolzano, Bolzano, Italy; 2 Medical Clinic V (Nephrology, Hypertensiology, Rheumatology, Endocrinology, Diabetology), Medical Faculty of Mannheim, University of Heidelberg, Mannheim, Germany; 3 Departement of Clinical Chemistry, University of Saarland, Homburg, Germany; 4 Medical University of Graz, Clinical Institute of Medical and Chemical Laboratory Diagnostics, Graz, Austria; 5 Synlab Academy, Synlab Holding Deutschland GmbH, Mannheim, Germany; Universitatsklinikum Hamburg-Eppendorf, GERMANY

## Abstract

**Introduction:**

Short telomeres have been associated with adverse lifestyle factors, cardiovascular risk factors and age-related diseases, including cardiovascular disease (CVD), myocardial infarction, atherosclerosis, hypertension, diabetes, and also with mortality. However, previous studies report conflicting results.

**Objectives:**

The aim of the present study has been to investigate the involvement of telomere length in all-cause and CVD mortality in subjects hospitalized for diagnostic coronary angiography of the Ludwigshafen Risk and Cardiovascular Health (LURIC) study.

**Methods:**

Relative telomere length (RTL) was measured with a Q-PCR based method in 3,316 participants of the LURIC study. Age-corrected RTL was calculated as the ratio between RTL and age. Median follow-up was 9.9 years. Cox regression and Kaplan-Maier analyses were performed to evaluate the role of RTL for all-cause and cardiovascular mortality.

**Results:**

RTL correlated negatively with age (r = -0.09; p<0.001). In surviving patients the correlation between age and RTL was statistically significant (r = -0.088; p<0.001), but not in patients who died during follow-up (r = -0.043; p = 0.20). Patients in quartiles 2–4 of RTL had a lower hazard ratio for all-cause mortality (HR:0.822; 95%CI 0.712–0.915; p = 0.008) and CVD-mortality (HR:0.836; 95%CI 0.722–0.969; p = 0.017) when compared to those in the 1^st^ quartile. Adjustment for major cardiovascular risk factors did not change this result, however additional adjustment for age attenuated this effect. Patients in the 4^th^ quartile of age-corrected RTL compared to those in the 1^st^ quartile had a lower hazard ratio for all-cause mortality, even with adjustment for major cardiovascular risk factors.

**Conclusions:**

The present study supports the hypothesis that short telomere length increases the risk of all-cause and CVD mortality. Age appears to be an important co-variate that explains a substantial fraction of this effect. It remains unclear whether short telomeres contribute directly to the increase in mortality or if they are simply a surrogate marker for other adverse processes of aging.

## Introduction

Aging is a major risk factor for the development of many common diseases, including cardiovascular disease (CVD), myocardial infarction (MI), stroke, hypertension, type 2 diabetes mellitus (T2DM), cancer, and chronic kidney disease (CKD) [1]. The prevalence of these diseases significantly increases with age, and they are major causes of frailty and death [1]. Telomeres are protective end caps of chromosomes, supporting genomic integrity and stability, key aspects of aging [1]. They are nucleoprotein structures composed by a non-coding, repetitive DNA sequence (TTAGGG) and associated proteins that form the shelterin complex [1]. Due to the inability of the DNA polymerase to fully replicate the 3’ end of chromosomes, telomeres progressively shorten with every cell division [1]. The consequence of this phenomenon is that a somatic cell can undergo a defined number of doublings before telomeres become critically short, lose their protective properties and send cells into senescence, or cause cell death [1]. Therefore, mean telomere length has been used as a biomarker of biological age [1]. Short telomeres have been associated with older age, adverse lifestyle factors, such as stress, smoking and obesity and reduced physical activity [1]. In addition, telomere length has been studied as a potential biomarker of age-related diseases, such as CVD [1,2–4], MI [1,2], atherosclerosis [1], hypertension [1,5], T2DM [1], and mortality [6–8]. In population-based prospective studies it has repeatedly been shown that individuals with short telomeres have an increased risk for cardiovascular events, stroke, MI and all-cause mortality [2,3,9,10]. Despite the increase in all-cause mortality, in the general elderly population short telomeres are not necessarily associated with a higher risk of cardiovascular death [6,9]. In studies that found a significant increase in cardiovascular mortality the effect was rather small [11] or limited to distinct sub-groups, such as African-American [10].

Large prospective studies that analyzed the relationship between telomere length and mortality in high-risk populations with pre-existing cardiovascular disease are missing. The present study aimed to fill this gap of knowledge investigating the association between leukocyte telomere length and all-cause as well as cardiovascular mortality in the participants hospitalized for diagnostic coronary angiography of the prospective Ludwigshafen Risk and Cardiovascular Health (LURIC) study. The LURIC study was designed to prospectively evaluate the effect of clinical and biochemical factors on cardiovascular outcomes.

## Methods

### Study cohort

A detailed description of the LURIC study has been published previously [12]. Briefly, 3,316 white patients hospitalized for elective diagnostic coronary angiography at the Heart Center Ludwigshafen (Germany) were enrolled between June 1997 and January 2000. Inclusion criteria were: German ancestry, clinical stability except for acute coronary syndromes and the availability of a coronary angiogram. Exclusion criteria were: any acute illness other than acute coronary syndromes, any chronic disease where non-cardiac disease predominated and a history of malignancies within the past five years.

All patients underwent a physical examination, coronary angiography and electrocardiography [12]. Coronary artery disease (CAD) was defined as a visible luminal narrowing of ≥20% stenosis in ≥1 of the 15 coronary segments [12]. The diagnosis of MI was either based on electrocardiographic criteria for ST elevation or non-ST elevation combined with chest pain for >20 minutes (being refractory to sublingual nitrates and/or typical enzyme elevations) or based on a report of a diagnosis of MI in a medical document [12]. T2DM was diagnosed according to the 2014 criteria of the American Diabetes Association. Moreover, patients with a history of diabetes and those using oral anti-diabetics or insulin were considered diabetic [12].

The study was approved by the ethics committee of the Physicians Chamber of Rheinland-Pfalz and performed in accordance with the declaration of Helsinki [12]. All participants gave written informed consent [12].

### Follow-up

Information about survival was obtained from local person registries. Two physicians blinded to baseline characteristics of the study participants classified causes of death by reviewing hospital records and death certificates. In the case of disagreement about classification, the final decision was made by one of the principal investigators of LURIC after appropriate review of the data [12]. Cardiovascular mortality was defined as death due to fatal MI, sudden cardiac death, death after cardiovascular intervention, stroke and other causes of death due to cardiovascular diseases [12]. The median follow-up time was 9.9 years (8.5–10.7).

### Laboratory measurements

Blood samples were collected in vacutainer tubes containing an anticoagulant (EDTA (ethylenediaminetetraacetate), citrate, or lithium heparin) or in tubes without anticoagulant. Laboratory measurements were assessed using standardized routine methods as described previously [12].

### Analysis of relative telomere length (RTL)

RTL was measured in genomic DNA using a quantitative-polymerase chain reaction (Q-PCR)-based assay [13]. Genomic DNA was extracted from whole blood by a standard salting-out procedure [12]. DNA quantity and quality was assessed by spectrophotometry analyzing absorbances at 230, 260 and 280 nm.

The master mix for each 20 μl PCR reaction was prepared with 4 μL LightCycler Fast Start DNA Master Plus SYBR Green I (Roche Diagnostics, Mannheim, Germany), 150 nmol/L of telomere-specific primers (TELO For, CGGTTTGTTTGGGTTTGGGTTTGGGTTTGGGTTTGGGTT and TELO Rev, GGCTTGCCTTACCCTTACCCTTACCCTTACCCTTACCCT), or 100 nmol/L of a single-copy housekeeping gene primers (36B4 For, CAGCAAGTGGGAAGGTGTAATCC and 36B4 Rev, CCCATTCTATCATCAACGGGTACAA). In each run 40 ng of sample DNA was analyzed in duplicate, a coefficient of variation (CV) between replicates of 2.5% was considered acceptable and the average of both replicates was calculated. All samples were run in duplicate and when the CV between the replicates was more than 2.5%, the measurement-replicates were repeated. The thermal cycling profile for both reactions consisted of a 95°C activation step, followed by 40 cycles of 95°C for 15 seconds and 58°C for 60 seconds. All Real-Time PCR reactions were carried out on a Lightcycler Instrument (Roche Diagnostics, Mannheim, Germany). A seven-point standard curve (dilution from 5 ng—100 ng) was run using a pool of 10 control DNAs for both the telomere and 36b4 PCRs to ensure linearity of the reaction (R^2^ >0.99). PCR efficiencies for telomeric sequence and reference gene were approximately equal, 2.1 and 1.94 respectively. The pooled control DNA was tested in all assays to allow comparability of the results. The CV between runs of the pooled control DNA was of 3.1% for the telomeric sequence and of 1.2% for the reference gene. DNA isolated from human embryonic kidney (HEK 293, Gibco, Karlsruhe, Germany) cells was used as reference control. The PCR data was analyzed with the comparative cycle threshold (Ct) method (2^-ΔΔCt^) [13]. Briefly, after calibration, the Ct of the telomeric sequence was subtracted by the Ct of the reference gene to calculate ΔCt. The ΔCt of the sample was subtracted from the ΔCt of the reference control to calculate the ΔΔCt. Finally, the relative telomere length (RTL) was calculated using the 2^-ΔΔCt^ equation. This method measures the relative expression of the telomeric sequence (telomere, T) in comparison to a reference gene (36b4, S). RTL analysis was performed at the Department of Clinical Chemistry and Laboratory Medicine at the University Hospital of the Saarland University in Homburg/Saar.

In order to consider the age dependent decline of telomere length we also performed an age-correction of RTL by calculating the RTL / age ratio.

### Statistical analyses

All data were examined for normality of their frequency distribution using the Kolmogrov-Smirnov test. Where indicated non-normally distributed variables were log-transformed prior to further statistical testing. Descriptive statistics provide means (±SD) or medians (10th-90th percentiles) for normally and non-normally distributed variables, respectively. Where indicated, quartiles of the entire study cohort were calculated. One-way ANOVA or a Kruskal-Wallis test were used to identify significant differences between multiple groups of continuous variables. The Mann-Whitney-U test was used to compare continuous variables between two independent groups. The correlation between RTL and age was calculated using the Pearson method. The Cox proportional hazard model was used to examine the association between quartiles of RTL and age-corrected RTL and time to death from any cause and from cardiovascular diseases. Adjustment for covariates was performed as indicated. Kaplan-Meier curves were produced to evaluate the cumulative survival during follow-up, according to quartiles of RTL and age-corrected RTL.

For the graphical presentation of results, box plots were used where the lines of each box represent the median, and the 25th and the 75th percentiles. All tests used were 2-sided and p values <0.05 were considered to be statistically significant.

All statistical analyses were performed using SPSS (Statistical Package for the Social Sciences, version 19.0) and R v3.4.1 (http://www.r-project.org). Hazard ratio plots were drawn using the R-package ‘rms’ (v5.1–1).

## Results

### RTL and baseline characteristics

In the LURIC cohort median RTL was 1.7881 (0.4651–4.9341). Table 1 and S1 Table show the baseline characteristics according to RTL and age-corrected RTL quartiles, respectively.

**Fig 1:**
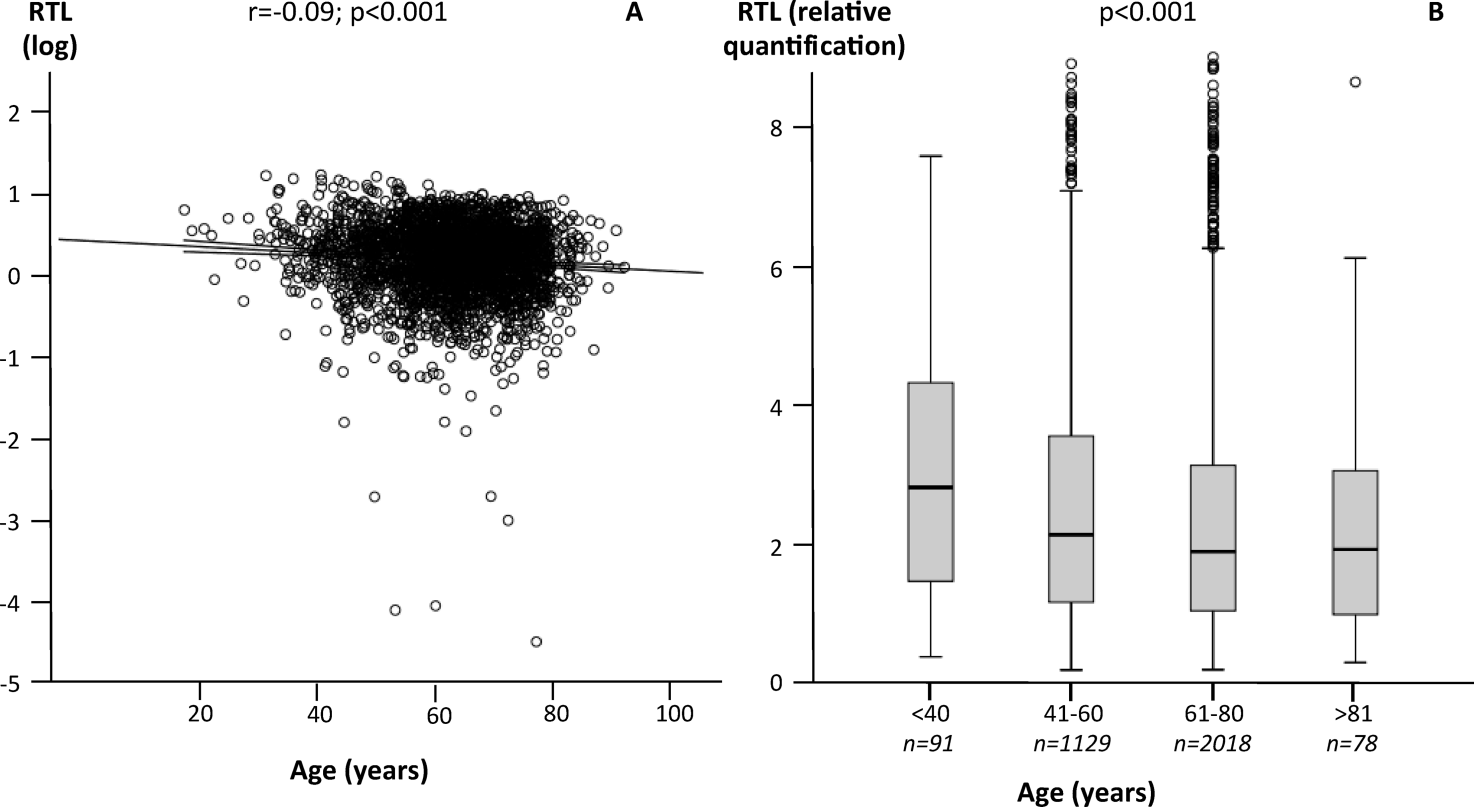
Associations between RTL and age. A) Correlation between RTL (log-transformed) and age. B) RTL according to age strata.

RTL correlated negatively with age (r = -0.09; p≤0.001; Fig 1A). Subjects below 40 years (median RTL 2.626) showed markedly longer telomeres compared to older subjects (p≤0.001). The median RTL was 1.9424 in individuals aged 41–60 years, 1.6953 in individuals aged 61–80 years, and 1.7451 in subjects older than 81 years (Fig 1B).

**Table 1:** Baseline characteristics according to RTL quartiles.

Parameter	All subjects	RTL quartiles				p-value of the trend	p-value 1^st^ vs all others
** **	** **	**1**^**st**^ **<0.8915**	**2**^**nd**^ **0.8916–1.7881**	**3**^**rd**^ **1.7882–3.1088**	**4**^**th**^ **>3.1089**	** **	** **
Age	62.7 ± 10.6	64.1 ±9.9	62.6 ±10.3	62.7 ±10.8	61.8 ±11.5	**<0.001**	**<0.001**
**No smoker (%)**	**36**	**36**	**34**	**36**	**37**	
**Ex smoker (%)**	**41**	**43**	**43**	**41**	**38**	**0.271**
**Active smoker (%)**	**23**	**22**	**23**	**23**	**25**	
BMI	27.1(22.9–32.72)	27.0(22.7–33.0)	27.1(22.9–33.2)	27.1(23.1–33.2)	27.0(22.6–32.1)	0.769	0.942
SBP (mmHg)	140(111–173)	142(114–177)	140(112–171)	140(110–173)	140(110–172)	0.054	**0.006**
DBP (mmHg)	81(66–96)	82(67–97)	80(67–95)	80(65–96)	81(66–96)	0.114	**0.016**
MHR (bpm)	67(55–84)	68(56–85)	68(56–83)	67(54–85)	67(54–84)	0.636	0.434
WBC (10^3^/nL)	6.76(4.80–9.80)	6.77(4.92–9.67)	6.81(4.91–9.90)	6.70(4.66–9.80)	6.70(4.73–9.80)	0.682	0.892
Hb (g/dL)	13.9(11.9–15.6)	13.8(11.7–15.6)	13.9(12.0–15.8)	13.8(11.9–15.6)	13.9(11.9–15.7)	0.310	0.278
Glucose (mg/dL)	102(88–154)	102(87–162)	103(88–153)	103(88–153)	101(87–146)	0.056	0.917
HbA1c (%)	6(5.2–7.9)	6(5.2–8.1)	6(5.3–7.9)	6(5.2–7.9)	5.9(5.2–7.5)	**0.012**	0.290
Creatinine (mg/dL)	0.9(0.7–1.2)	0.9(0.7–1.2)	0.9(0.7–1.2)	0.9(0.7–1.2)	0.9(0.7–1.2)	0.272	0.981
LDL (mg/dL)	114(75–159)	116(75–156)	117(78–160)	111(72–158)	112(77–158)	0.130	0.452
HDL (mg/dL)	37(26–53)	38(26–53)	37(27–54)	36(26–51)	38(26–53)	**0.007**	0.341
TnThs (pg/ml)	11(1.5–113)	11(1.5–180)	11(1.5–139)	10.5(1.5–109)	10(1.5–80)	0.082	**0.028**
NTproBNP (pg/ml)	293(46–2267)	319(53–2421)	283(47–2074)	285(46–2261)	296(38–2372)	0.122	**0.027**

BMI: body mass index; SBP: systolic blood pressure; DBP: diastolic blood pressure; MHR: mean heart rate; bpm: beats per minute; WBC: white blood cells; Hb: hemoglobin; HbA1c: glycosylated hemoglobin; LDL: low density lipoprotein; HDL: high density lipoprotein; TnThs: high sensitivity cardiac troponin T; NTproBNP: pro-B-type natriuretic peptide

RTL correlated negatively with SBP (r = -0.06; p = 0.001) and DBP (r = -0.039; p = 0.033), but neither with BMI (r = -0.007; p = 0.716), nor with the mean heart rate (r = -0.026; p = 0.16).

### RTL and mortality

Mean RTL was shorter in patients who died during follow up, compared to those alive (2.0405 vs. 2.2050; p = 0.015). Similar results were obtained using age-corrected RTL (mean age-corrected RTL 0.0426 vs. 0.0336; p<0.001). Two thousand three hundred twenty one patients survived and 995 died during follow-up.

**Fig 2:**
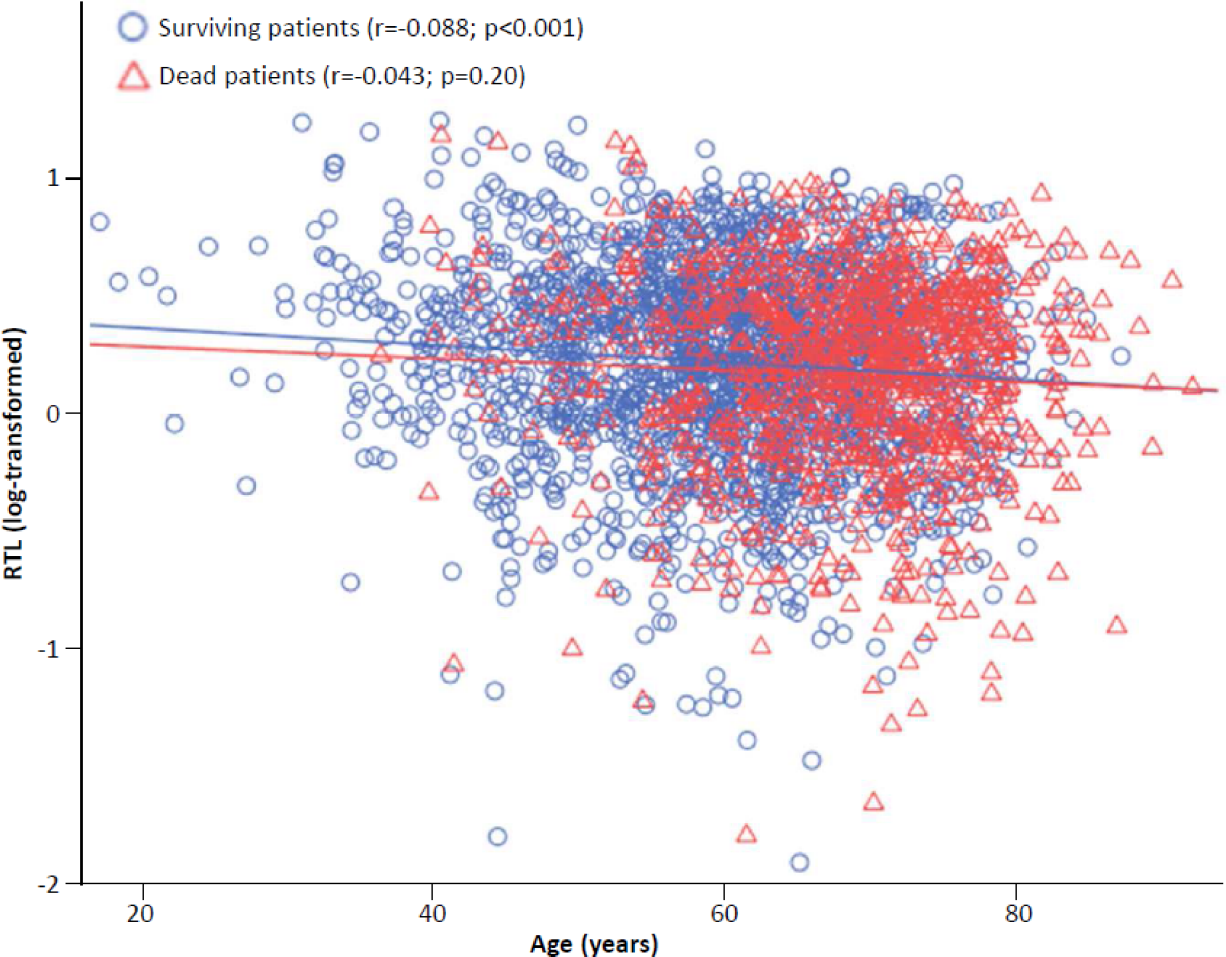
RTL as a function of age according to follow-up. Surviving patients are shown as blue circles (r = -0.088; p<0.001), deceased patients as red triangles (r = -0.043; p = 0.20).

Fig 2 shows log RTL plotted with age in patients who died or survived during follow-up. In surviving patients the correlation between age and RTL was statistically significant (r = -0.088; p<0.001), but not in patients who died during follow-up (r = -0.043; p = 0.20).

**Fig 3:**
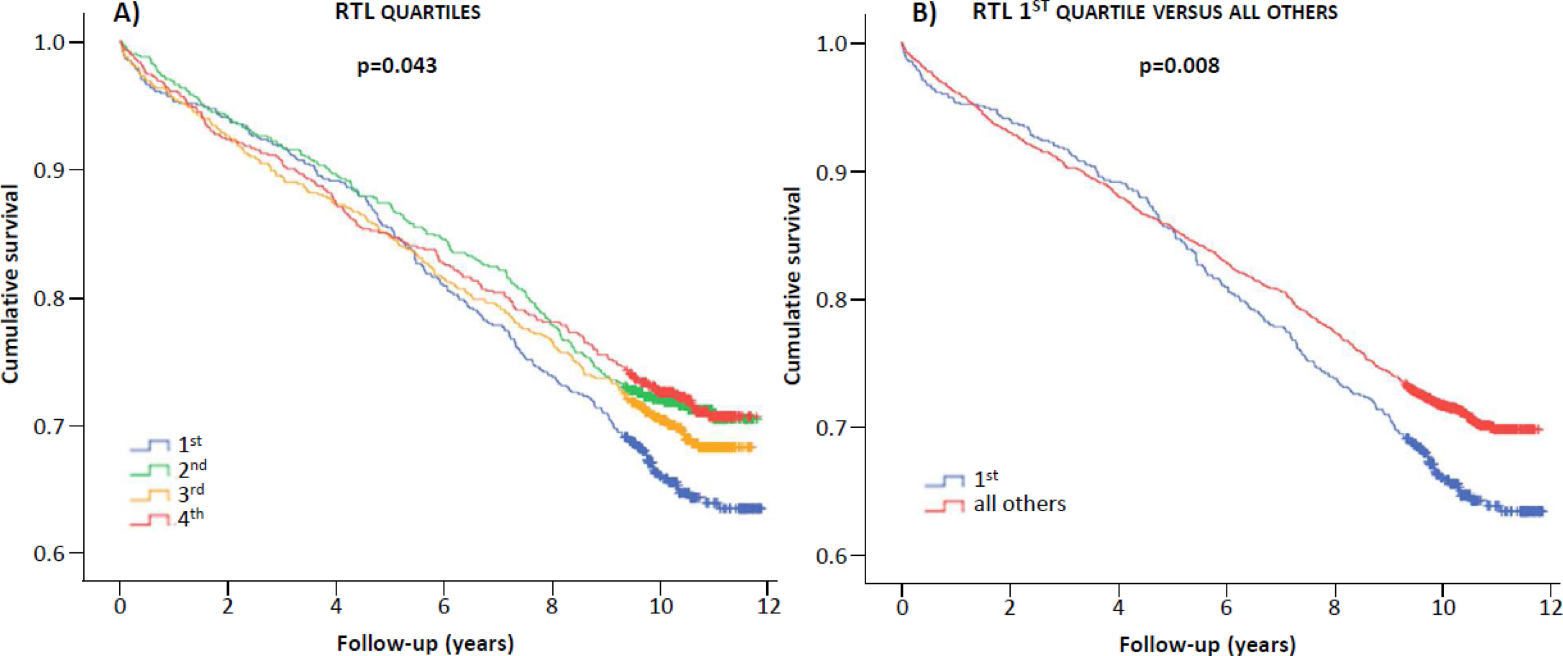
Kaplan-Meier plots. Cumulative survival according to RTL quartiles (A), RTL quartile 1 versus all others quartiles (B).

Fig 3 shows the cumulative survival rates according to quartiles of RTL. Patients in the 1^st^ quartile were characterized by lower survival compared to those in the other quartiles (p = 0.043, Fig 3A) or all others combined (p = 0.008, Fig 3B). Similar results were obtained using age-corrected RTL quartiles (S1 Fig).

The Cox regression for all-cause and CVD-mortality according to RTL quartiles shows that RTL is a predictor for CVD and all-cause mortality in the crude model as well as in the model adjusted for major cardiovascular risk factors, such as sex, LDL cholesterol, HDL cholesterol, triglycerides, body mass index, lipid lowering therapy, blood pressure, diabetes mellitus, smoking, CAD, high-sensitive C-reactive protein and estimated glomerular filtration rate (Table 2). However, further adjustment for age attenuated this result significantly. Furthermore, the Cox regression for all-cause and CVD-mortality according to age-corrected RTL quartiles (S2 Table) shows that age-corrected RTL is a predictor for CVD and all-cause mortality either in the crude model or with adjustment for major cardiovascular risk factors.

**Table 2:** Cox regression for all-cause and CVD-mortality according to RTL quartiles.

RTL quartiles	Model 1		Model 2		Model 3	
	HR (95% CI)	P	HR (95% CI)	P	HR (95% CI)	P
***All-cause mortality***						
1st (<0.8915)	Ref.		Ref.		Ref.	
2nd (0.8916–0.1.7881)	0.801 (0.668–0.962)	**0.017**	0.814 (0.678–0.977)	**0.028**	0.844 (0.702–1.013)	0.069
3rd (1.7882–3.1088)	0.873 (0.730–1.043)	0.135	0.854 (0.713–1.022)	0.085	0.877 (0.733–1.050)	0.154
4th (>3.1089)	0.794 (0.662–0.953)	**0.013**	0.861 (0.717–1.034)	0.108	0.931 (0.775–1.119)	0.447
2-3-4 (>0.8916)	0.822 (0.712–0.951)	**0.008**	0.842 (0.728–0.974)	**0.021**	0.882 (0.763–1.021)	0.092
***Cardiovascular mortality***						
1st (<0.8915)	Ref.		Ref.		Ref.	
2nd (0.8916–0.1.7881)	0.874 (0.695–1.100)	0.252	0.823 (0.684–0.991)	**0.040**	0.856 (0.711–1.030)	0.100
3rd (1.7882–3.1088)	0.892 (0.709–1.122)	0.328	0.864 (0.720–1.037)	0.116	0.890 (0.742–1.068)	0.211
4th (>3.1089)	0.841 (0.667–1.061)	0.143	0.878 (0.730–1.057)	0.170	0.951 (0.790–1.145)	0.598
2-3-4 (>0.8916)	0.836 (0.722–0.969)	**0.017**	0.855 (0.737–0.991)	**0.037**	0.897 (0.774–1.040)	0.150

Model 1: crude model. Model 2: adjusted for cardiovascular risk factors, such as sex, LDL-C, HDL-C, log(Triglyceride), BMI, lipid lowering therapy, blood pressure, diabetes, smoking, CAD, log(hsCRP), eGFR. Model 3: as model 2 further adjusted for age.

**Fig 4:**
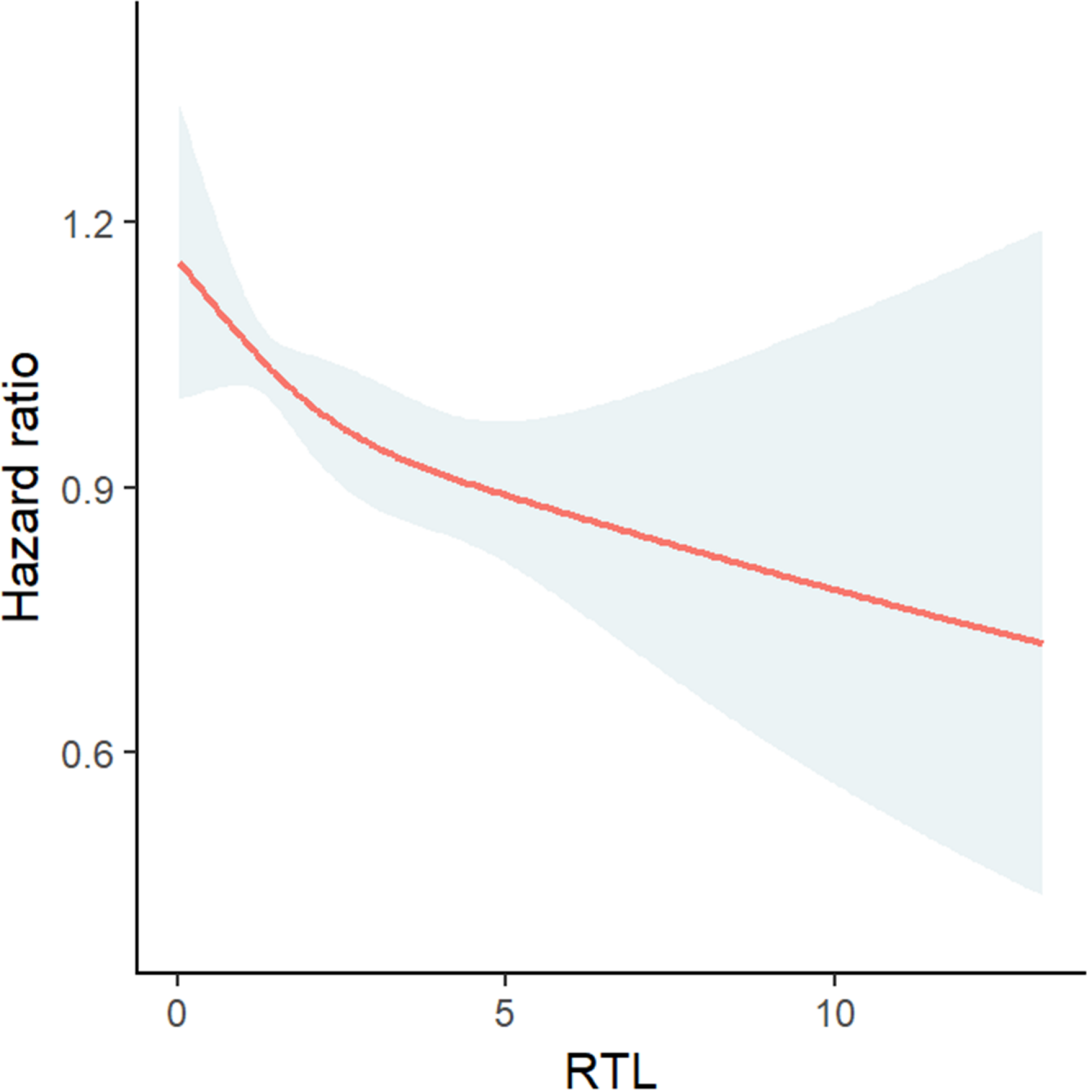
Relationship between RTL and all-cause mortality. RTL was modelled as restricted cubic spline in Cox regression analysis and plotted against the log relative hazard with 95% confidence intervals.

The relationship between RTL and age-corrected RTL and all-cause mortality are illustrated in Fig 4 and in S2 Fig, respectively.

### RTL and prevalent diseases

RTL was lower in patients with diabetes mellitus (p = 0.033) and CAD (p<0.001) compared to those without these conditions: median RTL was 1.69 in diabetics and 1.85 in non-diabetic subjects. Median RTL in CAD patients was 1.73 compared to 2.03 in CAD-free subjects.

## Discussion

This large-scale prospective study demonstrates that RTL is a predictor of all causes mortality and CVD mortality. Subjects with a RTL below the median of the cohort showed a reduced survival compared to those with RTL above the median. In addition, pre-existing diabetes mellitus or CAD were associated with shorter telomeres when compared to subjects without these conditions. However, traditional cardiovascular risk factors, in particular age, explain a substantial fraction of the association between RTL and mortality.

### Unselected or population based studies

Our results add substantial information to previous studies that have shown a relationship between RTL and mortality in healthy individuals or unselected populations [2,3,9,11,14,15]. For example, in the population-based Bruneck study, subjects with the longest telomeres had the lowest risk to develop CVD, stroke, MI and vascular death during a follow-up period of 10 years [2]. In the prospective WOSCOPS study that included 1,542 men with no history of MI, individuals with the shortest telomeres (1^st^ quartile) had a 44% higher incidence of CVD compared to individuals in the 4^th^ quartile [3]. In other large-scale studies subjects with the shortest telomeres had a 17–66% increase in mortality risk when compared to subjects with the longest telomeres [9,12,13]. Using data from four large Danish Studies including a total of 66,618 patients, Madrid et al. calculated a HR for incident ischemic heart disease during follow-up of 1.02 (95% CI, 1.01–1.03) for every 200-bp shorter telomere length [15]. However, all these studies investigated low-risk populations with a limited number of events whereas LURIC is a cohort of medium to high-risk cardiovascular patients. This may explain differences in effect size or the absence of significant effects. For example, in the Bruneck study only 88 subjects of the 800 randomly chosen individuals experienced a CVD event upon follow-up [2]. In 2,744 random selected elderly Swedish men RTL was neither related to all-cause mortality (HR 1.05; 95% CI 0.85–1.28) nor to CVD mortality (HR: 1.08; 95%CI 0.81–1.43) [8]. Similarly, Bischoff et al., did not find an association between telomere length and survival among 812 elderly subjects (HR: 0.97; 95%CI 0.83–1.14) from three different Danish cohorts [7]. However, the participants of these two studies were older than 75 years so that other protective factors may have overridden the potentially negative effects of short telomeres [7,8].

### Selected populations

Solid evidence supports a relationship between short telomeres and an increased risk of CVD [2,16,17]. A recent meta-analysis of 22,233 CAD cases and 64,762 controls from 21 population-based European cohorts showed that one standard deviation shorter telomeres increases CVD risk by 21% (95% CI 5%-35%) [17]. Moreover, a genome wide association study identified associations between CVD and several loci on genes involved in telomere biology including TERC, TERT, NaF1, OBFC1 and RTEL [17].

While the relationship between telomere length and CVD risk is well established there is limited knowledge about the impact of short telomeres on mortality in medium and high risk populations [4]. The few existing studies reported controversial results. In the MERIT-HF study telomere length was not related to all-cause mortality [4]. However, only 13 patients died during follow-up so that this study is probably underpowered [4].

The present results demonstrate 18 and 16% risk reduction in all-cause and CVD mortality, respectively, in patients with the longer telomeres when compared to those with the shortest telomeres. Adjustment for established cardiovascular risk factors attenuated these results. Additional adjustment for age further weakened the association between RTL and mortality and abolished significancy. However, using age-corrected RTL data, patients in the 4^th^ quartile compared to those in the 1^st^ quartile, had 42% and 40% risk reduction in all-cause and CVD mortality, respectively. Adjustment for common confounders did not change this result. Consequently, chronological age is a key factor that explains a significant fraction of RTL’s predictive power for mortality. The large, well-characterized study cohort and the long follow-up period confer substantial strength to our findings. Other studies in high-risk populations, such as the Cardiovascular Health Study (CHS) and the Lifestyle Interventions and Independence for Elders (LIFE) study, also demonstrated increased all-cause mortality in subjects with short telomeres [6,16]. However, the effect size ranges from 4–60% in these studies, which is probably explained by differences in study design, measurement of telomere length and different risk profiles of the study cohorts.

Also the change of telomere length in serial measurements seems to be related to mortality. For example, Goglin et al. analyzed RTL at baseline and after 5 years of follow-up in 954 subjects of the prospective Heart and Soul Study [18]. The variation of telomere length during this period was inversely associated with mortality. Every 325 bp increase in telomere change was associated with 36% lower risk of death (HR: 0.64; 95% CI, 0.54–0.76) [18]. In the LURIC study, no serial measurements of RTL were available so that we could not investigate this aspect.

### Mechanistic considerations

At present, it is not clear whether or not short telomeres contribute directly to CVD. Smoking, hypertension, diabetes mellitus are established CVD risk factors that are associated with reduced telomere length [1]. Therefore, the relationship between RTL and CVD mortality could simply reflect the adverse effects of these factors on vascular structure and function. However, most CVD risk factors induce oxidative stress and inflammation, both of which are known to accelerate telomere shortening. Short telomeres cause genetic instability and can induce cellular senescence and apoptosis. Therefore, a reduced RTL can promote senescence of endothelial cells and thus impair vessel function and repair [1]. In 2,165 American Indians of the Strong Heart Family Study Peng et al. reported an inverse association between telomere length and arterial stiffness [19]. Short telomeres are also related to a faster progression of atherosclerotic plaques [1] and the presence of unstable plaques in patients with acute coronary syndrome [1]. Other aspects, such as telomere uncapping with subsequent p53/p21-induced senescence may also influence vessel function and mortality risk [5].

### Analytical considerations

In the present study telomere length was assessed in blood leucocytes with a Q-PCR based method. This method determines the average relative telomere length of all chromosomes and all cells in a sample whereby telomere length is compared to a single copy reference gene. Q-PCR based methods are fast, highly sensitive, sufficiently cost-effective, allow high-throughput and require only small amounts of DNA. Although qPCR is an acceptable method for the assessment of telomere length, Southen blotting is considered the gold standard. Previous studies analyzing the correlation between both methods have reported a curvilinear relationship [20]. The larger measurement error of qPCR versus Southern blot could be a potential source of inconsistency in prior association studies with telomere length [20]. Other methods like quantitative fluorescence in situ hybridization (Q-FISH) and flow-cytometry (flow-FISH) provide additional information about the variability of telomere length between individual cells of the same sample. However, they require vital cells, which limits their application in large-scale epidemiological studies.

Another important aspect of the present study is the correction of RTL for age. The natural shortening of telomeres with age is modified by factors that accelerate or slow down this process. Consequently, the same RTL in a young and an old person has a different meaning. Age correction has been used in previous studies but there is no consensus on the correct approach [21,22]. For example, age-adjustment has been performed by subtracting the RTL at the 50th age-specific percentile in the study-cohort from the measured RTL of a sample [21]. Others have expressed age-adjusted RTL as z score [22]. In the present study, RTL was divided by age. This method better reflects the continuous decline of RTL with age, considers the characteristics of individual subjects and does not depend from the entire cohort. However, to ensure that age correction did not change the principle outcome of the study, we performed all tests before and after age-correction. Most effects were already visible when crude RTLs were used, but accentuated substantially after age correction.

## Conclusions

In conclusion, the present study supports the hypothesis that short telomere length increases the risk of all-cause and CVD mortality. However, a significant fraction of this association is explained by established cardiovascular risk factors, in particular chronologic age. It remains unclear whether short telomeres directly contribute to the increase in mortality or if they are simply a surrogate marker for other adverse processes of aging.

## Supporting information

S1 Table Baseline characteristics according to age-corrected RTL quartiles. (DOCX)

S2 Table Cox regression for all-cause and CVD-mortality according to age-corrected RTL quartiles. (DOCX)

S1 Fig Kaplan-Meier plots. Cumulative survival according to age-corrected RTL quartiles (A), age-corrected RTL quartile 1 versus all others quartiles (B).
(TIF)

S2 Fig Relationship between age-corrected RTL and all-cause mortality. Age-corrected RTL was modelled as restricted cubic spline in Cox regression analysis and plotted against the log relative hazard with 95% confidence intervals.
(TIF)
