# Supplementary material for: Telomere length and mortality in the Ludwigshafen Risk and Cardiovascular Health study
Source: PLoS One. 2018 Jun 19;13(6):e0198373. doi: 10.1371/journal.pone.0198373 (PMC6007915; doi:10.1371/journal.pone.0198373)
Supplement: S1 Table — (DOCX) [file pone.0198373.s001.docx]

| **S1 Table:** Baseline characteristics according to age-corrected RTL quartiles. | | | | | | |
| --- | --- | --- | --- | --- | --- | --- |
| **Parameter** | **Age-corrected RTL quartiles** | | | | p-value of the trend | p-value 4^th^ vs all others |
|  | **1^st^**  **<0.014** | **2^nd^**  **0.0141-0.028** | **3^rd^**  **0.0281-0.0509** | **4^th^**  **>0.0510** |  |  |
| Sex (M%) | 68 | 71 | 69 | 70 | 0.583 | 0.665 |
| Smoke (%)  No  Ex  Yes | 40  41  19 | 33  45  22 | 37  41  22 | 34  36  30 | **<0.001** | **<0.001** |
| SBP (mmHg) | 144 (115-177) | 141 (112-172) | 141 (111-174) | 136 (108-169) | **<0.001** | **<0.001** |
| DBP (mmHg) | 82 (67-97) | 80 (67-95) | 81 (66-97) | 80 (66-96) | **0.040** | 0.126 |
| MHR (bpm) | 67 (56-86) | 68 (56-84) | 67 (55-83) | 67 (54-85) | 0.400 | 0.136 |
| WBC (10^3^/nL) | 6.70 (4.87-9.65) | 6.85 (4.86-9.90) | 6.70 (4.80-9.60) | 6.74 (4.70-9.97) | 0.533 | 0.953 |
| Hb (g/dL) | 13.8 (11.7-15.5) | 13.9 (12.0-15.6) | 13.8 (12.0-15.7) | 14.0 (12.0-15.7) | **0.038** | **0.019** |
| Glucose (mg/dL) | 103 (87-162) | 103 (87-154) | 103 (87-156) | 101 (87-141) | **0.002** | **<0.001** |
| HbA1c (%) | 6.0 (5.2-8.2) | 6.0 (5.3-7.8) | 6.0 (5.2-7.9) | 5.9 (5.1-7.4) | **<0.001** | **<0.001** |
| Creatinine (mg/dL) | 0.9 (0.7-1.2) | 0.9 (0.7-1.2) | 0.9 (0.7-1.2) | 0.9 (0.7-1.2) | **0.005** | **<0.001** |
| LDL (mg/dL) | 116 (74-157) | 115 (76-159) | 111 (73-157) | 112 (78-158) | 0.450 | 0.902 |
| HDL (mg/dL) | 38 (26-54) | 37 (27-54) | 36 (26-51) | 37 (26-53) | 0.081 | 0.910 |
| TnThs (pg/ml) | 12 (1.5-163) | 11 (1.5-105) | 11 (3-105) | 9.2 (1.5-124) | **<0.001** | **<0.001** |
| NTproBNP (pg/ml) | 343 (64-2548) | 282 (46-2056) | 305 (49-2477) | 252 (33-2057) | **<0.001** | **<0.001** |

BMI: body mass index; bpm: beats per minute; CAD: coronary artery disease; CKD: chronic kidney disease; DBP: diastolic blood pressure; Hb: hemoglobin; HbA1c: glycosylated hemoglobin; HDL: high density lipoprotein; LDL: low density lipoprotein; MDRD: modification of diet in renal disease; MHR: mean heart rate; NTproBNP: pro-B-type natriuretic peptide; SBP: systolic blood pressure; TnThs: high sensitivity cardiac troponin T; WBC: white blood cells.
