# Supplementary material for: Telomere length and mortality in the Ludwigshafen Risk and Cardiovascular Health study
Source: PLoS One. 2018 Jun 19;13(6):e0198373. doi: 10.1371/journal.pone.0198373 (PMC6007915; doi:10.1371/journal.pone.0198373)
Supplement: S2 Table — (DOCX) [file pone.0198373.s002.docx]

| **S2 Table**: Cox regression for all-cause and CVD-mortality according to age-corrected RTL quartiles. | | | | |
| --- | --- | --- | --- | --- |
| Age-corrected RTL quartiles | Model 1 |  | Model 2 |  |
|  | HR (95% CI) | p | HR ( 95% CI) | p |
| ***All-cause mortality*** | | | | |
| 1st (<0.014) | Ref. |  |  |  |
| 2nd (0.0141-0.028) | 0.845 (0.710-1.006) | 0.058 | 0.877 (0.736-1.044) | 0.140 |
| 3rd (0.0281-0.0509) | 0.793 (0.664-0.964) | **0.010** | 0.792 (0.662-0.946) | **0.010** |
| 4th (>0.0510) | 0.579 (0.478-0.700) | **<0.001** | 0.729 (0.601-0.884) | **0.001** |
|  |  |  |  |  |
| ***Cardiovascular mortality*** | | | | |
| 1st (<0.014) | Ref. |  |  |  |
| 2nd (0.0141-0.028) | 0.856 (0.686-1.067) | 0.167 | 0.885 (0.709-1.106) | 0.283 |
| 3rd (0.0281-0.0509) | 0.799 (0.638-1.000) | **0.050** | 0.801 (0.639-1.004) | 0.054 |
| 4th (>0.0510) | 0.598 (0.470-0.760) | **<0.001** | 0.782 (0.613-0.997) | **0.047** |

Model 1: crude model. Model 2: model adjusted for cardiovascular risk factors, such as sex, LDL-C, HDL-C, log(Triglyceride), BMI, lipid lowering therapy, blood pressure, diabetes, smoking, CAD, log(hsCRP), eGFR. Ref.: reference
