# Supplementary figures and images for: Telomere length and mortality in the Ludwigshafen Risk and Cardiovascular Health study
Source: PLoS One. 2018 Jun 19;13(6):e0198373. doi: 10.1371/journal.pone.0198373 (PMC6007915; doi:10.1371/journal.pone.0198373)

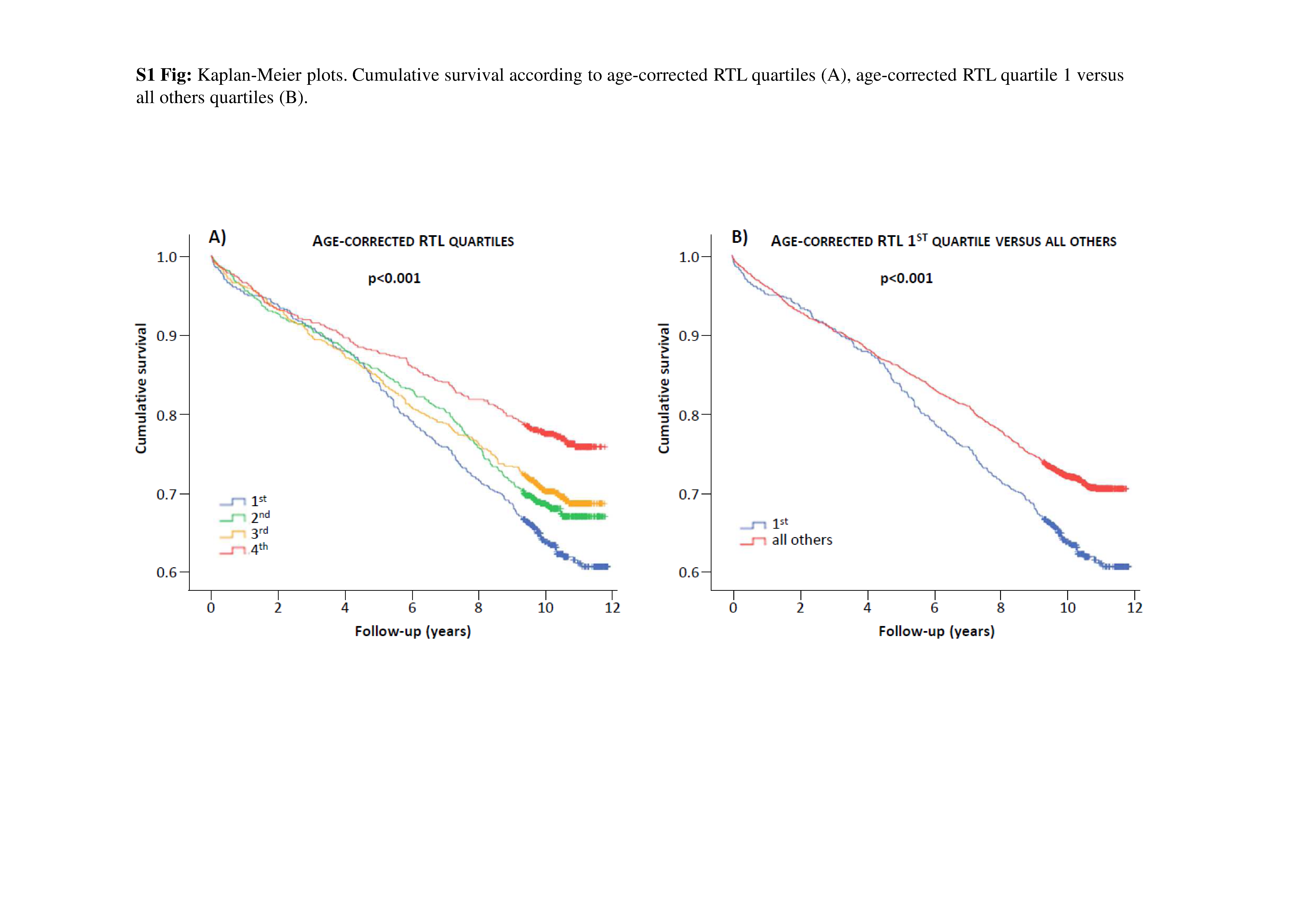

Supplement: S1 Fig — Cumulative survival according to age-corrected RTL quartiles (A), age-corrected RTL quartile 1 versus all others quartiles (B). (TIF) [file pone.0198373.s003.tif]

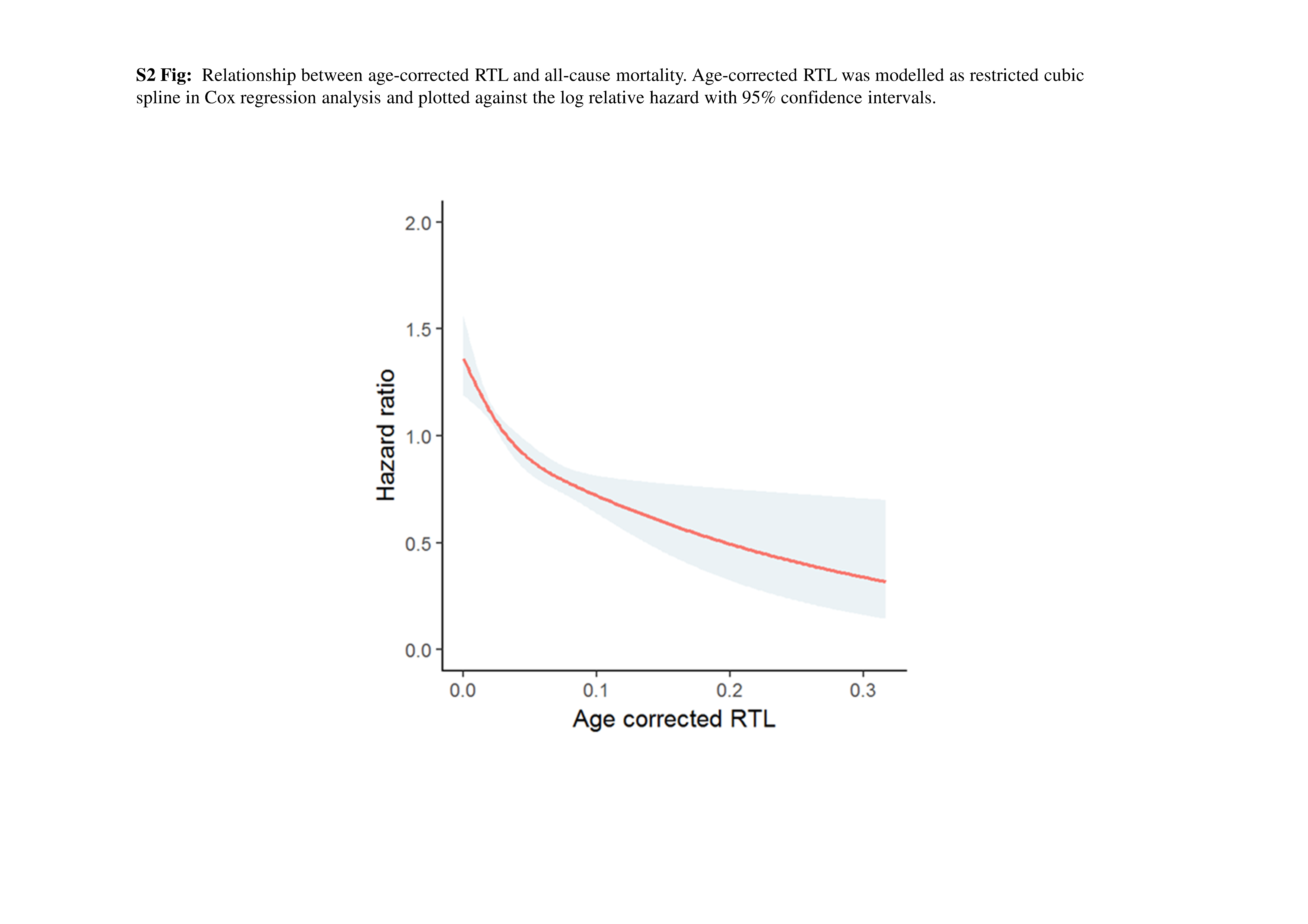

Supplement: S2 Fig — Age-corrected RTL was modelled as restricted cubic spline in Cox regression analysis and plotted against the log relative hazard with 95% confidence intervals. (TIF) [file pone.0198373.s004.tif]
